# Editorial: Cellular Senescence: Causes, Consequences and Therapeutic Opportunities

**DOI:** 10.3389/fcell.2022.884910

**Published:** 2022-03-23

**Authors:** Pooi-Fong Wong

**Affiliations:** Department of Pharmacology, Faculty of Medicine, Universiti Malaya, Kuala Lumpur, Malaysia

**Keywords:** ageing, cellular senescence, SASP, senescence markers, senotherapeutics

Once regarded as a tissue culture phenomenon, cellular senescence has long come of age. It is one of the hallmarks of aging and widely accepted as an important driver of the progression of age-related degenerative pathologies such as cardiovascular disease, neurodegenerative disease and type 2 diabetes (Muñoz-Espin and Serrano, 2014; Franceschi et al., 2018). The concept of reducing the burden of senescent cells or suppressing senescence-associate secretory phenotype (SASP) to delay age-related changes, improve tissue functions and subsequently increase health and life span were introduced and proven by many studies (Xu et al., 2015; Zhu et al., 2015, 2017; Chang et al., 2016; Fuhrmann-Stroissnigg et al., 2017). These ground-breaking findings underpin the rapidly expanding field of senotherapeutics to identify drugs that can eliminate senescent cells (senolytics) or suppress SASP (senomorphics) as a novel pharmacological approach to concurrently treat age-related multi-morbidities. In this Research Topic, we showcase both fundamental and translational advances in our understanding of cellular senescence, which will form the basis of future therapies for age-related degenerative changes. Schroth et al. discussed how senescent cell accumulation as a result of defective innate and adaptive immune responses leads to the progression of chronic kidney diseases (CKD), rationalizing the use of various immunomodulation approaches to enhance immunosurveillance and clearance of senescent cells for CKD treatment. Currently, strategies enhancing the cytotoxic activity of NK cells against senescent cells, activating the antibody-dependent cell-mediated cytotoxicity (ADCC) and dampening SASP-derived inflammatory milieu by targeting NF-κB and c/EBPβ transcriptional activities are being studied intensively (Paez-Ribes et al., 2019). Wilkinson and Hardman offered their new insight on the physiological and pathophysiological roles of cellular senescence in diabetic would healing. While short-lived tissue senescence is essential for effective repair, sustained tissue senescence involving the senescence-linked chemokine receptor, CXCR2, impairs diabetic wound healing. The blockage of CXCR2 promoted wound repair, thus, bolstering the notion of suppressing SASP for chronic wound healing treatment. Indeed, metformin, an effective anti-diabetic drug, via its SASP suppression activity has been shown to accelerate wound healing (Qing et al., 2019). Ramasamy et al. in their review on chondrocyte senescence and cartilage degeneration, highlighted the use of senolytics such as FOXO4-p53-interfering peptides or dasatinib/quercetin to selectively eliminate senescent cells during the *in vitro* expansion of cells used for autologous chondrocyte implantation (ACI). ACI is a targeted approach used to treat articular cartilage injuries and prevent the onset of post-traumatic osteoarthritis. Although effective in eliminating senescent cells within the expanded cell population and thus, increasing cell differentiation efficiency, whether these drugs could produce greater repair potential and promote cartilage repair clinically warrants further investigations (Huang et al., 2021). Similarly, Rayson et al. noted that not all geroprotectors produced expected clinical outcomes for osteoarthritis and osteoporosis. Although these compounds can alleviate pathologies following disease induction in animal models, they are in fact less effective in aged animals, suggesting that geroprotectors may be ineffective once the disease is well established. Metformin, for example, has been reported to attenuate cartilage degradation when administered in mice with early OA and in rhesus macaques exhibiting very early stage of cartilage damage (Rayson et al.).

To date, successful clinical applications of senotherapeutics remain limited by adverse effects and insufficient efficacy arising from the lack of selectivity and sensitivity of the current senotherapeutics. Ironing out these issues and optimization of dosing regimen would require extensive research and a thorough understanding on the mechanisms of cellular senescence. One of the challenges of senotherapeutics identification is to convincingly demonstrate senolytic or senomorphic effects *in vivo*. This is in view of cell-type specificity of senescence markers, SASP factors and senescent cell anti-apoptosis (SCAPs) mechanism. Alternative detection methods of senescent cells are needed to obtain reproducible and high quality *in vitro* and *in vivo* data. In his review on the different markers available for the detection of senescent cells, Faragher cautioned false positive arising from the timing of detection and deviations from the typical senescent cell physiology. In addition to SA-β-gal, visualisation of lipofuscin, ferritin and advanced glycation end products (AGEs) can be applied to reduce discrepancies in detection. Novel systems such as pigs and horses with higher clinical relevance to human disease and human progeria can be used to develop better detection techniques or drug screening models (Faragher). There are also challenges in differentiating quiescent and replicative senescent cells. Mehta et al. elegantly demonstrated the application of chromosome repositioning to differentiate senescence from quiescence and young from senescent cells. They observed that chromosome 10 relocation to the nuclear periphery was retarded in senescent cells. Chromosomal repositioning requires nuclear myosin 1β (NM1β). Its organization and distribution in the nucleoplasm were also altered in senescent cells, suggesting that NM1β could be a potential marker for senescence (Mehta et al.).

Targeting SASP with senomorphics could be a credible strategy to suppress the bystander effects of the senescent cell. Nonetheless, SASP is highly heterogenous and nonspecific, with its exact composition remains unknown since many different pathways are involved in SASP regulation. Kumari and Jat reviewed the different pathways in SASP regulation which converge to activate the transcription factors NF-κB and c/EBPβ in senescent cells. Identification of novel SASP mechanisms such as HMGA-NMPT-NAD^+^, cGAS-STING and NOTCH1 pathways may present new druggable candidates for novel senomorphic development. Whether these pathways are interconnected in driving SASP and the inflammatory milieu in age-related pathologies requires further investigations (Kumari and Jat). It is also critical to identify SASP factors that are distinct from inflammatory mediators commonly associated to many diseases. Till then, the use of SASP as an unequivocal cellular senescence marker remains restricted and may limit the therapeutic potentials of senomorphics.

In the last decade, the complexity of the mechanisms of cellular senescence is beginning to unravel. Beyond the classical pathways, p53/p21WAF1/CIP1 and p16INK4A/pRB tumor suppressor in mediating cell cycle arrest, recent research shows the involvement of the dimerization partner, retinoblastoma (RB)-like, E2 factor (E2F) and multi-vulval class B (DREAM) complex working in concert with p53 in repressing genes to halt cell cycle progression. Updates on the role of DREAM complex in cellular senescence is summarized by Kumari and Jat. Cell cycle arrest can be triggered by ribosomal DNA (rDNA) repeats instability by activating DNA damage response (DDR). The association of rDNA stability with cellular senescence and ageing has been reported in human, as evidenced by rDNA copy loss in senescent cells and blood from aged individuals (Ren et al., 2017). Lee and Ong provided an update on rDNA instability caused by the accumulation of extrachromosomal rDNA circles (ERCs) in yeasts. ERCs are derived from rDNA through intra-molecular homologous recombination of the chromosome. In yeast, ELL-associated factor 3 (Eaf3) was reported to promote rDNA instability, whereby its absence resulted in less ERCs accumulation (Morlot et al., 2019). Its human homolog, MORF4-related gene on chromosome 15 (MRG15) has also been reported to be involved in DNA repair, cell cycle progression and cellular senescence through the association with nuclear protein complexes, including RB (Chen et al., 2010). The updates provided by the two reviews have exemplified the added layers of complexity in cellular senescence mechanisms and that there are considerably more unknown layers remain to be uncovered. FoxOs are key regulators of longevity downstream of insulin and insulin-like growth factor signaling (IIS). Of the four mammalian FoxO isoforms (FoxO1, 3, 4 and 6), only FoxO3 has been consistently correlated with longevity in population studies and preclinical models (Martin et al., 2015). FoxOs are involved in autophagy and the regulation of the ubiquitin–proteasome system, yet the exact mechanism involved is still unclear. Kapetanou et al. demonstrated the role of FoxO1 in proteostasis maintenance by binding onto the promoter region of β5 proteasome subunit to regulate its expression in mice. FoxO1 knockout but not FoxO3 severely impairs proteasome activity in mice tissues. Knockdown of insulin receptor substrate 1 (IRS1) is known to enhance FoxO1 activity and they showed that depletion of IRS1 also enhances both the transcriptional activity of FoxO1 and proteasome function (Kapetanou et al.). Findings from their study has provided new evidence of FoxO1’s role in regulating proteosome function downstream of IIS.

Clearly from the above, there is still much more to learn about the mechanisms of cellular senescence and its pathophysiological implications. As research progresses, new modulators or druggable targets or pathways may be unveiled to offer novel intervention approached to delay age-related pathologies. Complete understanding the intricacy of cellular senescence and innovative drug delivery methods are warranted to address current issues related to drug specificity and sensitivity for considerations of the design of future therapeutics targeting senescence. Overall, the field of senescence and ageing is tremendously exciting with the possibility of finding drugs that can delay ageing-related pathologies but warrants caution in data interpretations and clinical applications.

## References

[B1] Chang J. Wang Y. Shao L. Laberge R.-M. Demaria M. Campisi J. (2016). Clearance of Senescent Cells by ABT263 Rejuvenates Aged Hematopoietic Stem Cells in Mice. Nat. Med. 22, 78–83. 10.1038/nm.4010 26657143 PMC4762215

[B2] Chen M. Tominaga K. Pereira-Smith O. M. (2010). Emerging Role of the MORF/MRG Gene Family in Various Biological Processes, Including Aging. Ann. N. Y Acad. Sci. 1197, 134–141. 10.1111/j.1749-6632.2010.05197.x 20536842 PMC2918256

[B3] Franceschi C. Garagnani P. Morsiani C. Conte M. Santoro A. Grignolio A. (2018). The Continuum of Aging and Age-Related Diseases: Common Mechanisms but Different Rates. Front. Med. 5, 61. 10.3389/fmed.2018.00061 PMC5890129 29662881

[B4] Fuhrmann-Stroissnigg H. Ling Y. Y. Zhao J. McGowan S. J. Zhu Y. Brooks R. W. (2017). Identification of HSP90 Inhibitors as a Novel Class of Senolytics. Nat. Commun. 8, 422. 10.1038/s41467-017-00314-z 28871086 PMC5583353

[B5] Huang Y. He Y. Makarcyzk M. J. Lin H. (2021). Senolytic Peptide FOXO4-DRI Selectively Removes Senescent Cells from *In Vitro* Expanded Human Chondrocytes. Front. Bioeng. Biotechnol. 9, 677576. 10.3389/fbioe.2021.677576 33996787 PMC8116695

[B6] Martins R. Lithgow G. J. Link W. (2015). Long Live FOXO : Unraveling the Role of FOXO Proteins in Aging and Longevity. Aging Cell 15, 196–207. 10.1111/acel.12427 26643314 PMC4783344

[B7] Morlot S. Song J. Léger-Silvestre I. Matifas A. Gadal O. Charvin G. (2019). Excessive rDNA Transcription Drives the Disruption in Nuclear Homeostasis during Entry into Senescence in Budding Yeast. Cel Rep. 28, 408–422. 10.1016/j.celrep.2019.06.032 31291577

[B8] Muñoz-Esp D. Serrano M. (2014). Cellular Senescence: from Physiology to Pathology. Nat. Rev. Mol. Cel. Biol. 15, 482–496. 10.1038/nrm3823 24954210

[B9] Paez‐Ribes M. González‐Gualda E. Doherty G. J. Muñoz‐Espín D. (2019). Targeting Senescent Cells in Translational Medicine. EMBO Mol. Med. 11, e10234. 10.15252/emmm.201810234 31746100 PMC6895604

[B10] Qing L. Fu J. Wu P. Zhou Z. Yu F. Tang J. (2019). Metformin Induces the M2 Macrophage Polarization to Accelerate the Wound Healing via Regulating AMPK/mTOR/NLRP3 Inflammasome Singling Pathway. Am. J. Transl. Res. 11 (2), 655–668. 30899369 PMC6413292

[B11] Ren R. Deng L. Xue Y. Suzuki K. Zhang W. Yu Y. (2017). Visualization of Aging-Associated Chromatin Alterations with an Engineered TALE System. Cell Res 27, 483–504. 10.1038/cr.2017.18 28139645 PMC5385610

[B12] Xu M. Tchkonia T. Ding H. Ogrodnik M. Lubbers E. R. Pirtskhalava T. (2015). JAK Inhibition Alleviates the Cellular Senescence-Associated Secretory Phenotype and Frailty in Old Age. Proc. Natl. Acad. Sci. U.S.A. 112, E6301–E6310. 10.1073/pnas.1515386112 26578790 PMC4655580

[B13] Zhu Y. Doornebal E. J. Pirtskhalava T. Giorgadze N. Wentworth M. Fuhrmann-Stroissnigg H. (2017). New Agents that Target Senescent Cells: the Flavone, Fisetin, and the BCL-XL Inhibitors, A1331852 and A1155463. Aging 9, 955–963. 10.18632/aging.101202 28273655 PMC5391241

[B14] Zhu Y. Tchkonia T. Pirtskhalava T. Gower A. C. Ding H. Giorgadze N. (2015). The Achilles' Heel of Senescent Cells: from Transcriptome to Senolytic Drugs. Aging Cell 14, 644–658. 10.1111/acel.12344 25754370 PMC4531078

